# Nuclear Export of Human Hepatitis B Virus Core Protein and Pregenomic RNA Depends on the Cellular NXF1-p15 Machinery

**DOI:** 10.1371/journal.pone.0106683

**Published:** 2014-10-31

**Authors:** Ching-Chun Yang, Er-Yi Huang, Hung-Cheng Li, Pei-Yi Su, Chiaho Shih

**Affiliations:** 1 Taiwan International Graduate Program (TIGP) in Molecular Medicine, National Yang-Ming University and Academia Sinica, Taipei, Taiwan; 2 Institute of Biomedical Sciences, Academia Sinica, Taipei, Taiwan; University of Toronto, Canada

## Abstract

Hepatitis B virus (HBV) core protein (HBc) can shuttle between nucleus and cytoplasm. Cytoplasm-predominant HBc is clinically associated with severe liver inflammation. Previously, we found that HBc arginine-rich domain (ARD) can associate with a host factor NXF1 (TAP) by coimmunoprecipitation. It is well known that NXF1-p15 heterodimer can serve as a major export receptor of nuclear mRNA as a ribonucleoprotein complex (RNP). In the NXF1-p15 pathway, TREX (transcription/export) complex plays an important role in coupling nuclear pre-mRNA processing with mRNA export in mammalian cells. Here, we tested the hypothesis whether HBc and HBV specific RNA can be exported via the TREX and NXF1-p15 mediated pathway. We demonstrated here that HBc can physically and specifically associate with TREX components, and the NXF1-p15 export receptor by coimmunoprecipitation. Accumulation of HBc protein in the nucleus can be induced by the interference with TREX and NXF1-p15 mediated RNA export machinery. HBV transcripts encodes a non-spliced 3.5 kb pregenomic RNA (pgRNA) which can serve as a template for reverse transcription. Cytoplasmic HBV pgRNA appeared to be reduced by siRNA treatment specific for the NXF1-p15 complex by quantitative RT-qPCR and Northern blot analyses. This result suggests that the pgRNA was also exported via the NXF1-p15 machinery. We entertain the hypothesis that HBc protein can be exported as an RNP cargo via the mRNA export pathway by hijacking the TREX and NXF1-p15 complex. In our current and previous studies, HBc is not required for pgRNA accumulation in the cytoplasm. Furthermore, HBc ARD can mediate nuclear export of a chimeric protein containing HBc ARD in a pgRNA-independent manner. Taken together, it suggests that while both pgRNA and HBc protein exports are dependent on NXF1-p15, they are using the same export machinery in a manner independent of each other.

## Introduction

Hepatitis B virus (HBV) is one of the most common infectious agents worldwide [1], [2]. Despite the fact that HBV vaccine is successful, chronic HBV infection is often not curable, albeit treatable. HBV is the smallest DNA animal virus with a genome size near 3.2 kb [3]. An HBV genome encodes a multi-functional core protein (HBc) which can form capsid particles for the reverse transcription of HBV pregenomic RNA (pgRNA) [4], and interact with envelope protein in virion secretion [5]–[7].

Biogenesis of eukaryotic RNA occurs in the nucleus. Many viruses can take advantage of the host’s nuclear machinery for the production of their own viral RNAs. These nuclear RNAs usually need to be assembled into a ribonucleoprotein (RNP) complex for further processing and export to the cytoplasm via either the CRM-1 (XPO1) or the NXF1-p15 (TAP-NXT1) dependent pathway [8], [9]. Human CRM-1 is well known for its role in the export of non-spliced RNAs of HIV-1 [10], [11], foamy virus [12], and adenoviral early mRNA [13]. In the NXF1-p15 pathway, TREX (transcription/export) complex was proposed to couple nuclear pre-mRNA processing with mRNA export [14]. Examples for the NXF1-p15 export pathway include herpes simplex virus type 1 [15], Epstein-Barr virus [16], and murine leukemia virus [17]. Taken together, different viruses can take either the NXF1 or CRM-1 dependent pathway for RNA export.

**Figure 1:**
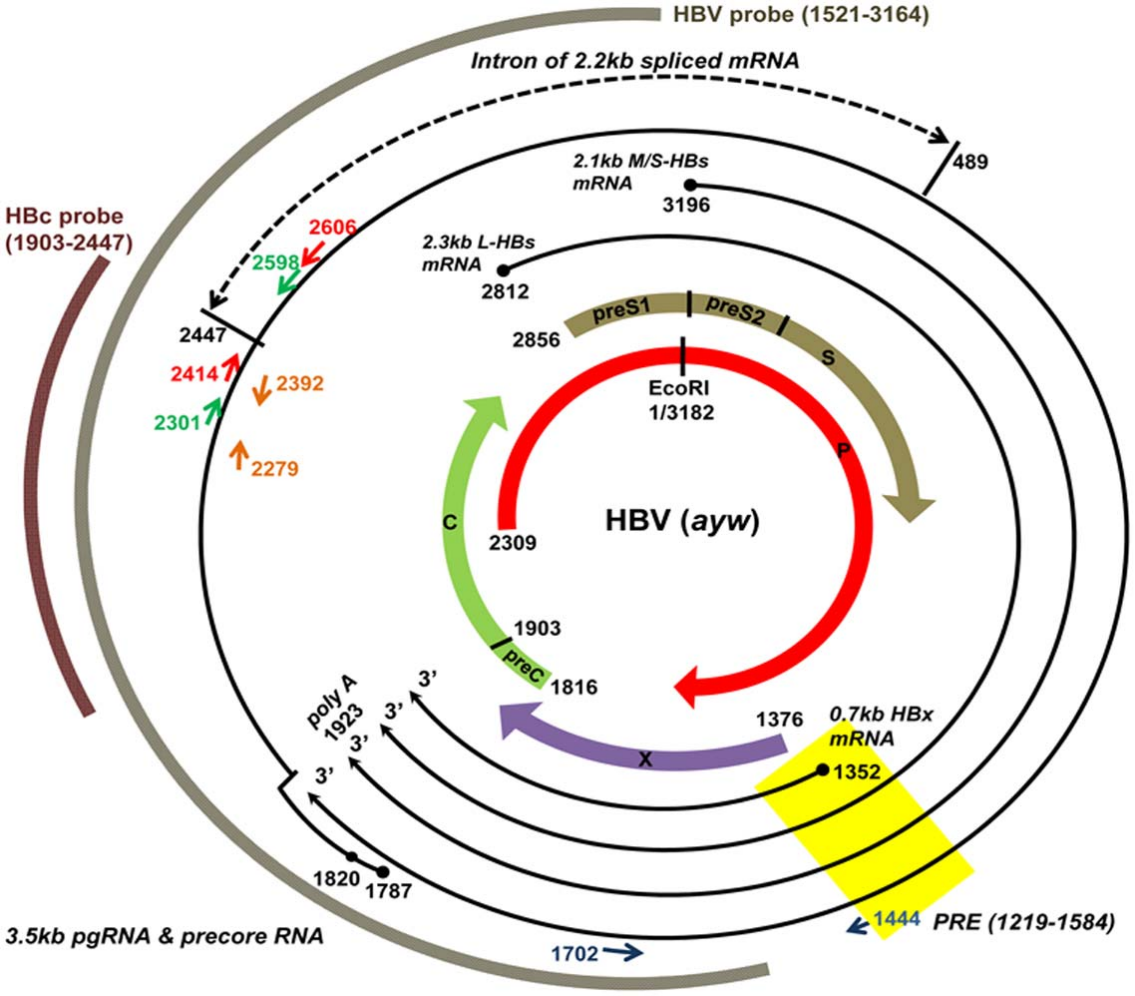
An HBV transcription map is illustrated. All HBV (*ayw*) transcripts are shown in solid lines, and a major intron of HBV pgRNA is shown in a dashed line (HBV: 2447–489). PCR primers used for detection of all species of HBV RNAs are shown in blue (HBV: 1444–1702), and primers used for non-spliced pgRNA are shown in red and green (HBV: 2414–2606, HBV: 2301–2598). PCR primers used for detection of HBV core^+^ RNAs are shown in brown (HBV: 2279–2392). An HBV genome is known to encode a 3.5 kb precore specific RNA which is responsible for the production of precore and HBeAg proteins. This 3.5 kb precore transcript is longer than the 3.5 kb pgRNA by 25–30 nt at the 5′ terminus [45]. The primer pairs HBV 2301–2598 and 2414–2606 cannot distinguish between the precore RNA and pgRNA. However, our HBV replicon system (plasmid pCHT-9/3091) does not produce the 3.5 kb precore RNA [30]. Core^+^ RNAs refer to the 3.5 kb pgRNA, 3.5 kb precore RNA, and the 2.2 kb major spliced RNA. HBc probe (nt 1903–2447) in Northern blot analysis was specific for HBV core^+^ RNAs. HBV probe (nt 1521–3164) was prepared for Southern blot analysis of viral replication. C: HBV core, preC: HBV precore, P: polymerase, preS1, preS2, and S: HBV envelope, X: HBx. PRE: posttranscriptional regulatory element [19].

Unlike the aforementioned large DNA viruses, HBV is the smallest DNA animal virus with a genome size near 3.2 kb [3], [18]. As shown in Fig. 1, major non-spliced HBV RNA transcripts include the 3.5 kb pgRNA, 3.5 kb precore RNA, 2.3 kb/2.1 kb HBV surface antigen (HBsAg) RNAs, and 0.7 kb HBx specific RNA. There are two important functions of the 3.5 kb pgRNA. One is to serve as a template of reverse transcription for an HBV genome, and the other is as an mRNA template for translation of polymerase and core protein. Previously, nuclear export of non-spliced HBsAg specific RNAs had been actively investigated. A RNA *cis*-element, so-called post-transcriptional regulatory element (PRE), was proposed to be important for the nuclear export of HBsAg specific RNAs [19]–[24]. In contrast to HBsAg specific RNAs, very little has been studied regarding how pgRNA and spliced RNAs are exported. Among the multiple HBV spliced transcripts, the most predominant HBV spliced RNA species has a size around 2.2 kb (Fig. 1) [25].

Recently, we demonstrated that HBc is a nucleocytoplasmic shuttling protein, containing both nuclear localization (NLS) and nuclear export signals (NES) [26]. Clinically, disease severity of chronic hepatitis B patients is associated with cytoplasmic HBc [27], [28], which is a major target of immune attack. A better understanding of HBc nuclear export could help understand better acute exacerbation or spontaneous relapse in HBV carriers. To date, it remains unclear how nuclear HBc protein gets exported.

To address this issue, we examined the physical interactions among HBV pgRNA, HBc, TREX and NXF1-p15 complex. In addition, we examined the functional effects of TREX and NXF1-p15 complex on the subcellular distribution of HBc protein and HBV pgRNAs in cell culture. Our results lend a strong support for an important role of the NXF1-p15 pathway for the nuclear export of an HBV ribonucleoprotein (RNP) complex, which consists of HBV core protein and RNA. Perturbation of HBV nuclear RNA export could have a therapeutic potential.

## Materials and Methods

### Cell culture and transfection

Human hepatoma cell line HuH-7 was maintained as described previously [26]. Lipofectamine 2000 (*Invitrogen, USA*) was used for co-transfection of siRNAs and plasmid DNA. PolyJet DNA is an in vitro tranfection reagent (*SignaGen, USA*) for plasmid DNA only (without siRNA).

### Plasmids

Plasmid K128T is an NLS-deficient mutant of SV40 large T antigen (SV40 LT). The chimera plasmid K128T-HBc ARD was cloned by inserting a PCR fragment of HBc ARD with a linker (gly-gly-ser-gly-gly-ser) and KpnI/EcoRI sites into the C-terminus of K128T. K128T lost its nuclear localization signal (NLS) due to the K128T mutation and does not contain any nuclear export signal (NES) either [26], [29]. Plasmid p15-Flag was cloned by inserting a PCR fragment of p15 with EcoRI/BamHI sites into pRK5-Flag vector. Plasmid pCHT-9/3091 is an HBV (*ayw*) 1.2 mer replication construct without precore and HBeAg [30]. Plasmid pCMV-Rluc-HBV was cloned by inserting a PCR fragment of renilla luciferase with EcoRI sites into the major intron of an HBV genome pCHT-9/3091. Plasmid ΔHBc was derived from pCHT-9/3091 by creating a stop codon at amino acid 16 of HBc via QuickChange XL Site-Directed Mutagenesis (*Stratagene Co, USA*). Plasmids HBc NLS and NES mutants were derived from an HBV replicon plasmid pCHT-9/3091 with arginine-to-alanine substitutions at HBc ARD-I and -III, as well as HBc ARD-II and ARD-IV, respectively [26]. pCMV-HBc 183 was cloned by inserting a PCR fragment containing HBc 183 open reading frame (ORF) into pcDNA 3.1 (+) vector at BamHI/EcoRI sites. Plasmids pCMV-ALY, pCMV-DDX39, pCMV-BAT1-Flag were purchased from *OriGene, USA*.

### Antibodies

IgG control (mouse or rabbit), anti-SVLT, anti-tubulin and anti-BAT1/DDX39 antibodies were purchased from *ICON- GeneTex, Taiwan*. Anti- streptavidin HRP were purchased from *Invitrogen, USA*. Anti-ALY, anti-CRM-1, and anti-Flag antibodies were purchased from *Santa Cruz, USA.* Anti-NXF1 was from *Sigma-Aldrich, USA*. Anti-GST was from *GeneCopoeia, USA.* Anti-HBc antibodies used in this study were purchased from *Dako, Denmark,* and *Institute of Immunology, Japan* (Hyb-3120) or from our own preparation [26].

### Co-immunoprecipitation (co-IP)

Transfected HuH-7 cells were lysed with IP buffer (20 mM Tris pH 8.0, 120 mM NaCl, 0.2% NP-40, 1 mM EDTA, 50 mM NaF, and 1 mM Na3VO4) in the presence of protease inhibitors *(Calbiochem, USA)*. Immunoprecipitation was performed by using specific antibodies with or without RNase A (250 ug/ml, *Fermentas, USA*) at 37°C for 20 min, followed by incubation with Dynabeads protein A or G (*Invitrogen, USA*) [26].

### Streptavidin Pull down Assay

Biotin-HBc ARD peptide was synthesized from *Yao-Hong Biotechnology, Taiwan*. Purified GST-NXF1 and GST-p15 proteins were purchased from *Abnova, Taiwan*. 100 nM biotin-HBc ARD polypeptide was incubated with 150 ng of purified GST-NXF1 and/or 150 ng of GST-p15 proteins in 200 µl final volume of IP buffer at 4°C for 2 hrs. An aliquot of 20 µl out of the 200 µl final volume was used as the input control. Dynabeads streptavidin T1 *(Invitrogen, USA)* was used for precipitation of the biotinylated peptide and its associated proteins. Proteins were eluted from beads by sample loading buffer. Standard procedures of Western blot were performed as described elsewhere [26].

### GST Pull down Assay

GST and GST-HBc ARD proteins were expressed in *E. coli* and purified by glutathione agarose beads (*Sigma-Aldrich, USA*) according to the vendor’s protocol. Equal amounts of GST and GST-HBc ARD proteins on the beads, as judged in a separate SDS-PAGE, were each incubated with 0.5–1 mg of HuH-7 cell lysates in IP buffer at 4°C for at least 2 hrs. Proteins were eluted from beads by reduced glutathione (*Sigma-Aldrich, USA*). 15–20 µg of cell lysates were used as an input control. Anti-ALY and anti-GST antibodies were used in Western blot assay.

### Immunofluorescence analysis (IFA)

IFA was performed as described in detail elsewhere [26]. One major drawback of the conventional IFA is in its qualitative or semi-quantitative nature in the measurement of changes in subcellular localization. To circumvent this deficiency, we invented a new method for data processing. We first divided all the antibody staining-positive cells into three groups: N>C (nucleus-predominant), N+C (both nucleus and cytoplasm), and C>N (cytoplasm-predominant). The conventional method of IFA is to measure the percentage of N>C pattern *only,* as an index of the tendency of nuclear accumulation of that particular protein under study. However, it is very common that the tendency of nuclear accumulation can often be detected as well by the shifting from pattern C>N into pattern C+N, but not necessarily into pattern N>C. To improve the quantitative analysis of IFA, we invented the measurement of the tendency of nuclear accumulation by scoring the ratio N>C/C>N. The *accuracy* and *sensitivity* of detecting changes in subcellular localization can be significantly improved by scoring the ratio N>C/C>N, rather than by scoring simply the percentage of N>C.

### HBV luciferase reporter assay, Southern blot analysis and MTT assay

HuH-7 cells were harvested 36–48 hrs after co-transfection with pCMV-Rluc-HBV plasmid and specific siRNAs. Luciferase signals were measured by Dual-Luciferase reporter assay *(Promega, USA)*. Nuclear export of many cellular RNAs is known to be mediated by the NXF1-p15 pathway [8], [9]. Therefore, perturbations of this pathway by siRNA treatments specific for NXF1, p15, ALY, or any other involved host factors, could affect the RNA export of both viral and cellular RNAs. To control for the indirect effect on HBV RNA export from these affected cellular RNAs, we routinely included a firefly luciferase plasmid as an internal control for normalization of the global effect from siRNA knockdown. Southern blot analysis was performed as described previously [31], [32] by using an HBV probe (Fig. 1). The MTT assay was conducted according to the vendor’s protocol [26].

### Northern blot and RT- qPCR analysis of fractionated RNA samples

RNA samples from HuH-7 cells were extracted from total cell lysate (C+N), nuclear (N) and cytoplasmic (C) fractions (RNeasy mini kit, *Qiagen, Germany*). Equal amounts of RNA from each fraction were loaded onto 1% formaldehyde agarose gel. Standard Northern blot procedure was performed with an HBc specific probe (Fig. 1). 18S and 28S rRNAs were stained with Healthview nucleic acid stain (*Genomics, Taiwan*). RNA samples were pretreated with amplification grade-DNase I *(Sigma-Aldrich, USA)* before reverse transcription (High Capacity cDNA Reverse Transcription Kits, *Applied Biosystems, USA*). Standard protocol of SYBR green qPCR system *(Applied Biosystems, 7500)* was performed [31]. GAPDH mRNA or snRNA U1 were included as an internal control for RT-qPCR.

### RNA immunoprecipitation (RNA-IP) assay

This protocol was adapted from literature [33]. Briefly, HuH-7 cells were harvested at 48 hours post-transfection. Formaldehyde was added (to 1% final concentration) to allow crosslinking for 10 minutes. To quench crosslinking, glycine was added to a final concentration of 250 mM, and cells were washed with PBS. Pelleted cells were lysed in IP buffer with RiboLock RNase Inhibitor *(Fermentas, USA)* and sonicated briefly. The protein concentration of cell lysates was measured by Bio-Rad protein assay *(Bio-Rad, USA)*. Aliquots of protein (1 mg) were used for immunoprecipitation. A 1/10 aliquot was preserved as an input sample and kept frozen at −80°C until a later step of reverse crosslinking. Antibodies were added to each sample and incubated at 4°C overnight. For the collection of immunoprecipitated complexes, 30 µl of Dynabead protein A/G beads were added to each sample with a continuous slow mixing rotation for more than 2 hours. Each immune complex sample was then washed five times with IP buffer, before incubation with 50 µl elution buffer (20 mM Tris-HCl pH 8.0, 1 mM EDTA, 1% SDS) at 37°C for 10 min. For reverse crosslinking, proteinase K *(Merck, USA)* was added to 1 µg/µl final concentration, and allowed incubation at 50°C for 1 hr, followed by 70°C for 1 hour. RNAs were extracted from the reverse crosslinked samples according to the manufacturer’s instructions (RNeasy mini kit, *Qiagen, Germany*). DNA contamination was removed by DNase I treatment as described above. RNAs were detected by standard RT-qPCR protocol using PCR primers as shown in Fig. 1.

### RT-qPCR primers

The qPCR primer pairs designed by NCBI programs were as follows (sequence of 5′-3′): HBV core^+^ RNAs (HBV-2279-F: TTCGCACTCCTCCAGCTTAT, HBV-2392-R: GAGGCGAGGGAGTTCTTCTT); HBV non-spliced pgRNAs (HBV-2414-F: CGCGTCGCAGAAGATCTCAA, HBV-2606-R: TGAGTGGGCCTACAAACTGT; HBV-2301-F: ACCACCAAATGCCCCTATCC, HBV-2598-R: AACTGTGAGTGGGCCTACAA); HBV all species RNAs (HBV-1444-F: GAAGAATTCGAATCCTGCGGACGACCC, HBV-1702-R: CTCAAGGTCGGTCGTTGACA); snRNA U1 (F: ACCTGGCAGGGGAGATACCA, R: GGGGAAAGCGCGAACGCAGT); p15 (F: GGGAACAAACAACGGGACTTCA, R: GGAAGCAGTCACTTGCGATC); UIF (F: CCTGAAGGTGCAGGCCCAGTTG, R: TGGTGGAAGTCCGCCATTGACGA); GAPDH (F: CAACTACATGGTTTACATGTTC, R: GCCAGTGGACTCCACGAC).

CRM-1 (F: TGATCCACAGATGGTCGCTG, R: CTGGTTCTCTAGCAGCTGGG); pri-miR-122 (F: GTTTCCTTAGCAGAGCTGTGGA, R: GCCAGCCTAGCAGTAGCTATTT).

### Knockdown by siRNAs

Lipofectamine 2000 (*Invitrogen, USA*) was used for co-transfection of siRNAs and plasmid DNA [26]. Cells were harvested less than 48 hours post-transfection for IFA, Northern blot, and RT-qPCR analyses. The siRNAs were purchased from *(Dharmacon, USA)*. Targeted sequences of specific genes are as follows: TAP/NXF1 gene (ON-TARGETplus SMARTpool siRNA, target sequence (5′ to 3′): GGGAAGUCGUACAGCGAAC; GCGCCAUUCGCGAACGAUU; AAUUGAAGUCUGAGCGGGA; CGAUGAUGAACGCGUUAAU); p15/NXT1 gene (ON-TARGETplus SMARTpool siRNA, target sequence: CAACGGGACUUCAACCAGA; GUUCCAAAUCAGCGUGGUA; GGUCCUUGUUGUCAUCUGU; GGUCUGGAAUGGCAAUGCU); ALY/THOC4 gene (ON-TARGETplus SMARTpool siRNA, target sequence: UCUCAGACGCCGAUAUUCA; GUUAAACAGACCAGCAAAU; GGAACUCUUUGCUGAAUUU; CAAAACAACUUCCCGACAA); BAT1/UAP56 gene (ON-TARGETplus SMARTpool siRNA, target sequence: GUAGAAGACUCGCCCAUUU; GGGCUUGGCUAUCACAUUU; GAAUGGAUGUCCUGUGCCA; GAACUGCCCGCAUAUCGUC); DDX39 gene (ON-TARGETplus SMARTpool siRNA, target sequence: AGGUGAUAAUCUUCGUCAA; CAUCGAGCAGAGCCGGUAA; ACAGUGAGAAGAACCGCAA; UGGAGGUGUUUGUGGACGA); UIF/FYTTD1 gene (ON-TARGETplus SMARTpool siRNA, target sequence: ACAUAAACAGUGUCGGAAA; GCAAAGAGAACUCGUCAAU; GAAGAGUUCAUCACGGAAA; AGACAGGGAUGACGUUGAA); CRM1/XPO1 gene (ON-TARGETplus SMARTpool siRNA, target sequence: AAGAAUGGCUCAAGAAGUA, GGACAAGAGUCGACACAAU, UAGAUAAUGUGGUGAAUUG, CGAAAUGUCUCUCUGAAGU). SiNonTarget was used as a siRNA knock down control.

### Accession Numbers

Accession numbers (Entrez Gene/UniProtKB and Swiss-Prot) for human TAP(NXF1) sequences are 10482/Q9UBU9, for human p15 (NXT1) are 29107/Q9UKK6, for human ALY (REF, THOC4) are 10189/Q86V81, for human UIF (FYTTD1) are 84248/Q96QD9, for human DDX39 (DDX39a) are 10212/O00148, for human BAT1 (DDX39b, UAP56) are 7919/Q13838, for SV40LT are 1489531/P03070, for human GAPDH are 2597/P04406, and hepatitis B virus (*ayw*) is V01460 in GenBank.

## Results

In our previous studies, we demonstrated that HBc protein can be physically associated with a cellular factor NXF1 (TAP) protein by co-immunoprecipitation (co-IP) [26]. The biological significance of this phenomenon remains unclear. To investigate the mechanism of nuclear export of HBc, we asked whether HBc can also interact with other protein factors of the NXF1-p15 export receptor machinery.

### Full-length HBc and HBc arginine rich domain (ARD) can associate with p15 in a ribonuclease sensitive manner

**Figure 2:**
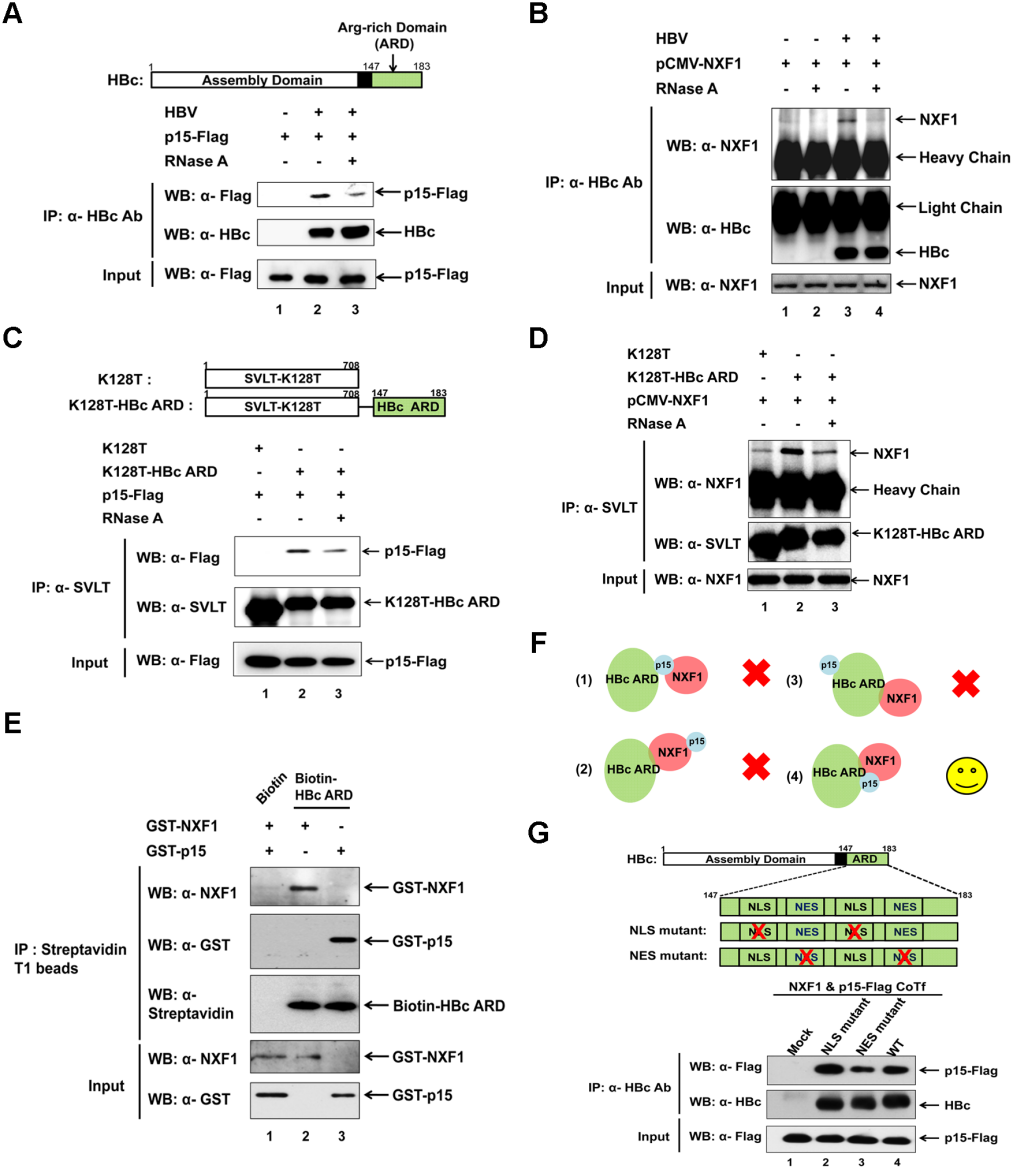
HBV core protein (HBc) can physically and specifically associate with the cellular NXF1-p15 complex via the NES (nuclear export signal) motif of HBc arginine rich domain (ARD). **A)** *Upper panel*: Full-length HBc (HBc 183) consists of a capsid assembly domain and an arginine rich domain (amino acid 147–183). *Lower panel:* HuH-7 cells were co-transfected with an HBV genome and a p15-Flag expression vector. Full-length HBc 183 protein can physically and specifically associate with a p15-Flag protein in a ribonuclease (RNase)-sensitive manner by coimmunoprecipitation assay (co-IP) and Western blot analysis (WB). **B)** Full-length HBc 183 protein was shown to associate with the native exogenous NXF1 protein by the same co-IP assay as described above. **C)** The *upper panel* shows a schematic outline of a K128T-HBc ARD chimera construct. The rationale of the experimental design is as detailed in the text. The co-IP result here demonstrated that only the chimera protein K128T-HBc ARD, but not K128T, can physically associate with the p15-Flag protein. **D)** By the same co-IP assay, only the chimera protein K128T-HBc ARD can physically associate with the native exogenous NXF1 in an RNase sensitive manner. **E)** Biotin-HBc ARD synthetic polypeptide can *in* *vitro* pull down purified recombinant proteins of GST-NXF1 and GST-p15 by using streptavidin T1 beads (Materials and Methods). **F)** A cartoon illustrates that either NXF1 or p15 can bind directly to HBc ARD. Possibility 3 refers to a situation when NXF1 and p15 do not form heterodimer even at high concentrations. The results in Fig. 2E support for possibility 4. **G)** *Upper panel:* A schematic outline of the mapped NLS and NES of HBc ARD [26]. *Lower panel:* HuH-7 cells were co-transfected (CoTf) with plasmid DNAs of NXF1 and p15-Flag expression vectors, and a mutant HBV genome containing R-to-A mutations at HBc NES or NLS subdomains. This NES mutant HBc exhibited significantly decreased physical association with the p15 protein (compare lane 3 with lanes 2 and 4). Ablation of the two NLS motifs appeared to increase slightly the intensity of p15-Flag (compare lane 2 and 4), suggesting that the wild type NLS could have a moderate occlusion effect on the association between p15-Flag and their neighboring NES.

It is known that NXF1 and p15 can form a heterodimer [34], [35]. When HuH-7 cells were co-transfected with an HBV genome and p15-Flag or native NXF1 expression vector, full-length HBc can specifically associate with p15 (Fig. 2A) or NXF1 (Fig. 2B) protein by co-IP assay. In our co-IP experiments, both p15 and NXF1 were provided from exogenous source by transfection, since the endogenous levels of these two proteins in HuH-7 cells were not always high enough for the co-IP assay. We used p15-Flag as a substitute to the native p15, because our anti-Flag antibody provided us a very robust detection of the p15-Flag protein in the co-IP experiments. To further map the interaction domain of full-length HBc with p15 or NXF1, we constructed a chimera protein by fusing only the HBc ARD in-frame with a reporter protein of mutant SV40 large T antigen (SVLT-K128T) (Fig. 2C, *upper panel*). Wild type SVLT is known to contain a potent NLS [29], but without any NES. Mutation K128T inactivated this NLS of SVLT by substitution from lysine to threonine at amino acid 128. Wild type HBc can self-assemble into a 240-mer icosahedral particle with most ARD domains buried in the capsid interior [36]. In the context of this chimera protein of K128T-HBc ARD, both NLS and NES of the HBc ARD domain will no longer be buried in the capsid interior due to the lack of the capsid assembly domain [37] (*Upper panel*, Fig. 2A, 2C). We demonstrated here that this chimera protein K128T-HBc ARD can also specifically associate with both p15-Flag (Fig. 2C) and NXF1 (Fig. 2D) by co-IP. In contrast, the control protein K128T, without HBc ARD, can bring down only a marginal amount of p15-Flag (Fig. 2C) and NXF1 (Fig. 2D). The physical associations between HBc ARD and NXF1 or p15-Flag were significantly diminished upon treatment with ribonuclease (RNase) in the co-IP assay (lane 3 of Fig. 2A, 2C, 2D; lane 4, Fig. 2B), suggesting that RNA can contribute to the binding between HBc ARD and the NXF1-p15 complex.

In addition to the co-IP experiment, we also examined the interactions between HBc ARD and the NXF1-p15 complex by the streptavidin pull down assay (Fig. 2E). The purified recombinant protein of GST-NXF1 (Materials and Methods), when incubated with a biotin-HBc ARD polypeptide in the absence of p15, can be pull down by using the streptavidin beads (lane 2, Fig. 2E). Similarly, the purified recombinant protein of GST-p15 (Materials and Methods), when incubated with a biotin-HBc ARD polypeptide in the absence of NXF1, can be pull down by using the streptavidin beads (lane 3, Fig. 2E). When RNA or RNase was added to the pull down assay, we observed no apparent effect on the results of the pull down assay (data not shown). Therefore, although RNA could enhance or contribute to the protein-protein association between HBc, NXF1, and p15 in the co-IP experiment (Fig. 2A–2D), it is not absolutely essential for the protein-protein interaction in the cell-free pull down assay (Fig. 2E).

As shown in Fig. 2F, we cartoon illustrated four hypothetical physical relationships among HBc, NXF1 and p15 proteins. The first possibility in Fig. 2F can be excluded by the fact that GST-NXF1 can interact with HBc-ARD in the absence of p15 (lane 2, Fig. 2E). Similarly, the second possibility can be excluded by the fact that GST-p15 can interact with HBc-ARD polypeptide in an NXF1-independent manner (lane 3, Fig. 2E). We consider possibility 3 as highly unlikely since it is well known that NXF1 and p15 can form a heterodimer [9]. By elimination of possibilities 1–3 in Fig. 2F, we are more in favor of possibility 4 that either protein can interact with the other two proteins directly.

As shown in Fig. 2G, HBc ARD contains two NLS and two NES [26]. To further fine map the subdomain of HBc ARD associated with NXF1-p15 complex, we performed co-IP experiment using HBc NLS and NES mutants. Preferential immunoprecipitation of p15-Flag was observed with the HBc NLS mutant (lane 2, Fig. 2G) over the HBc NES mutant (lane 3, Fig. 2G) in a highly reproducible manner. Unlike the conventional leucine-rich CRM-1 dependent NES, which typically interacts with karyopherins to mediate nuclear export of proteins, our result here suggests that nuclear export of HBc protein could be mediated by the association between arginine-rich HBc NES and the NXF1-p15 RNA export machinery.

### Full-length HBc and HBc ARD can physically associate with TREX

**Figure 3:**
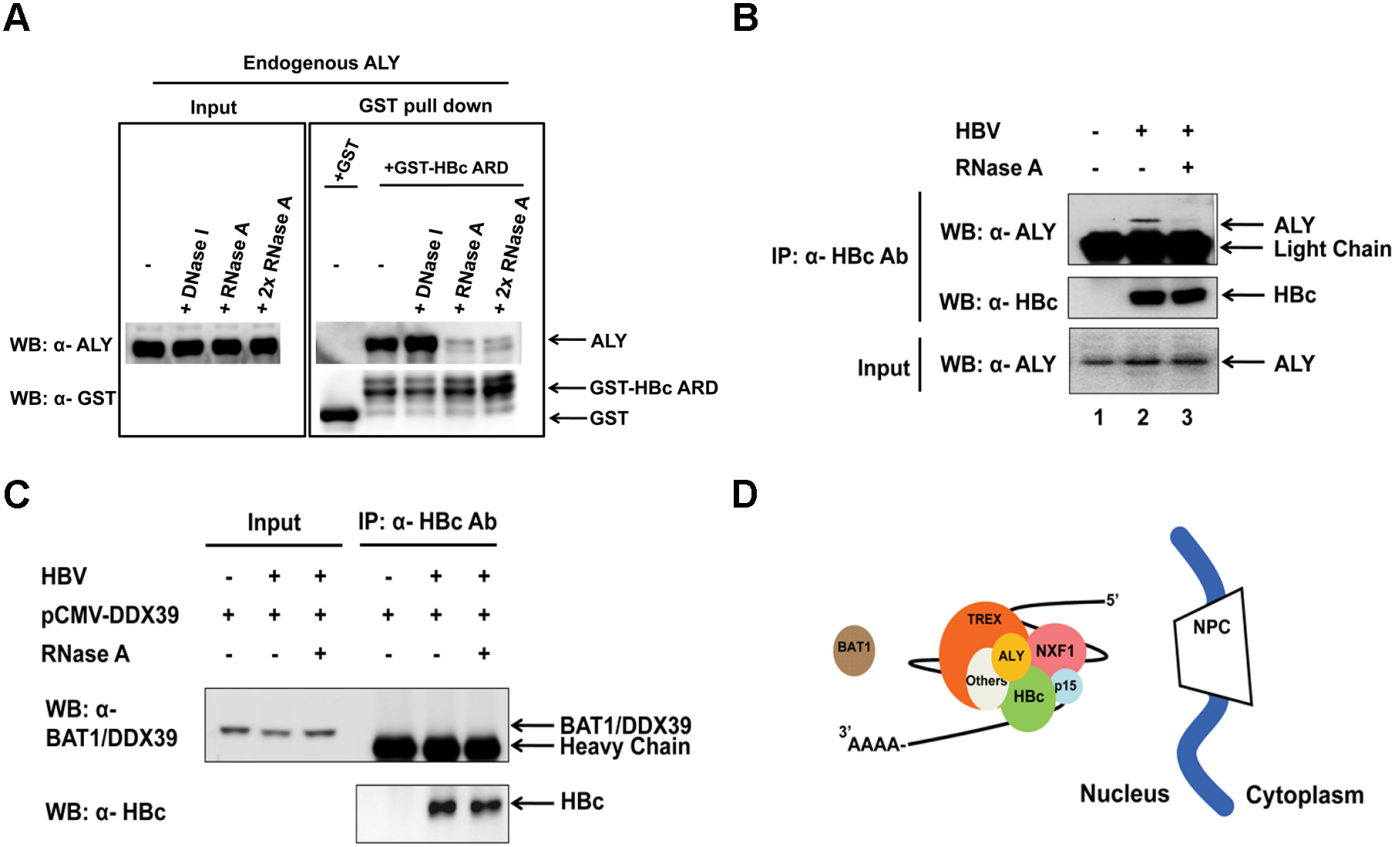
Association between HBc ARD and TREX complex. **A)** The association between GST-HBc ARD and a TREX component ALY was ribonuclease (RNase)-sensitive and DNase-resistant in a GST pull down assay. This assay was performed by using *E. coli -*expressed GST or GST-HBc ARD protein and untransfected HuH-7 cell lysates (Materials and Methods). **B)** Full-length HBc 183 protein in HuH-7 cells transiently transfected with an HBV genome can also co-immunoprecipitate the endogenous ALY in an RNase-sensitive manner. **C)** HuH-7 cells were co-transfected with an HBV genome and a pCMV-DDX39 expression vector. Full-length HBc 183 protein cannot associate with another TREX component BAT1/DDX39 in the co-IP assay. **D)** A cartoon summarizes the putative associations among HBc, RNA, ALY, TREX components, and others (other known or unknown cellular factors). Such associations are postulated to be involved in nuclear RNA processing and export. NPC: nuclear pore complex.

As mentioned in the Introduction, a so-called TREX complex can couple the pre-mRNA processing and nuclear export events [14]. Having demonstrated that HBc ARD could associate with the NXF1-p15 complex (Fig. 2), we next asked whether HBc ARD can bind to any known TREX components. Using a GST pull down assay (Materials and Methods; Fig. 3A), we demonstrated that the recombinant chimera protein of GST-HBc ARD can pull down a cellular RNA binding adaptor ALY, which is a TREX component known to be involved in the NXF1-p15 RNA export pathway [38]. Furthermore, the association between ALY and HBc ARD was RNase-sensitive and DNase-resistant (Fig. 3A). By co-IP experiment, we also demonstrated that full-length HBc protein can associate with the endogenous ALY in an RNase-sensitive manner (Fig. 3B). However, unlike ALY, HBc cannot associate with a cellular RNA helicase BAT1 (UAP56)/DDX39 (URH49) (Fig. 3C), which is also known to be involved in the NXF1-p15 mediated RNA export pathway [39]. The cartoon of Fig. 3D summarizes a hypothetical RNP complex of HBc, RNA, and various host factors, such as TREX components in the nucleus, which presumably can be involved in RNA splicing, trafficking and export.

### Nuclear export of HBc protein depends on TREX and NXF1-p15 complex

**Figure 4:**
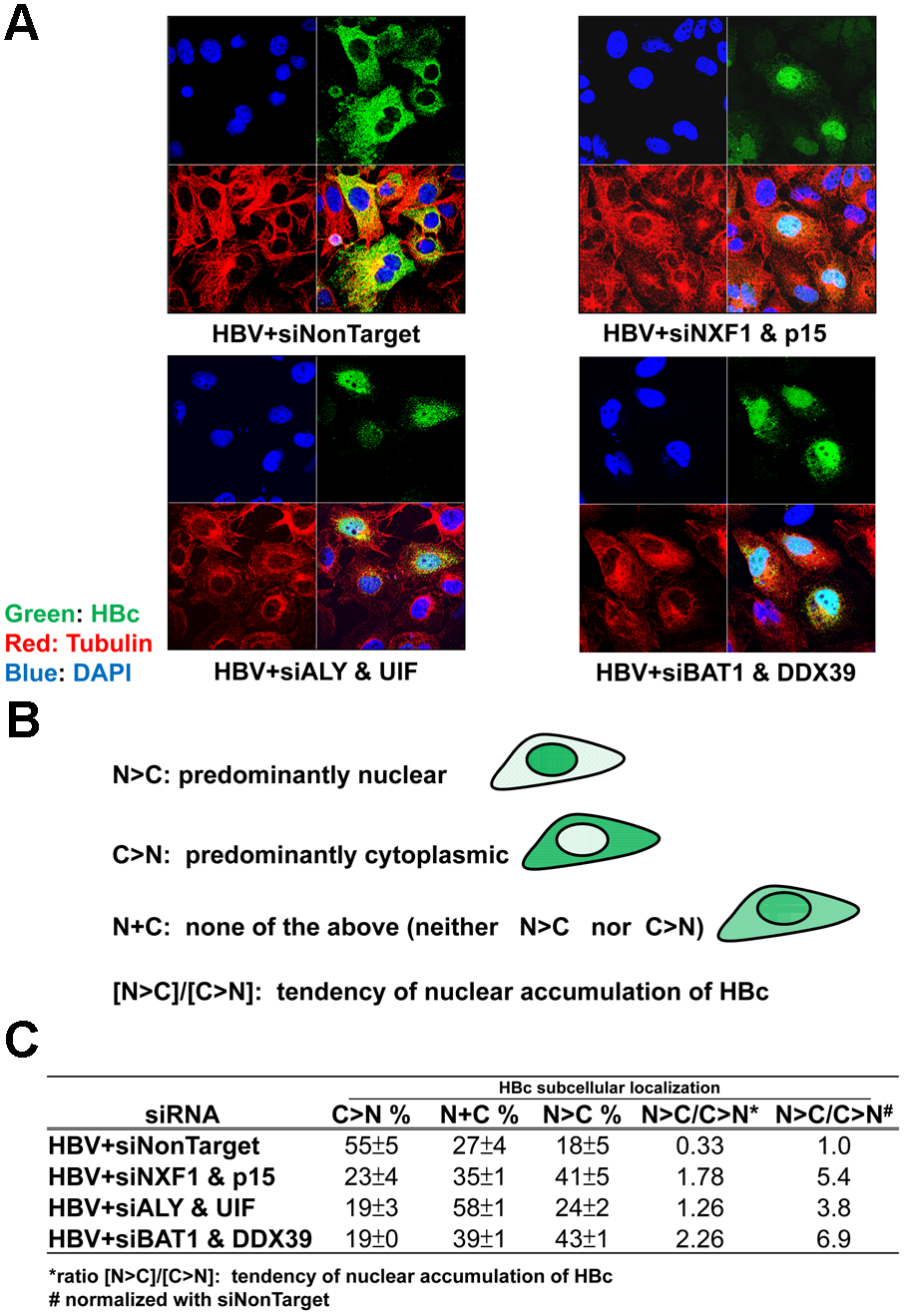
Perturbation of the TREX complex and NXF1-p15 nuclear export machinery by siRNA treatments can result in accumulation of HBc protein in the nucleus by IFA. **A)** An HBV genome and siRNAs were co-transfected into HuH-7 cells. Transfected culture was fixed 40 hrs post-transfection before IFA. A negative control by siNonTarget treatment exhibited a cytoplasmic predominant pattern (C>N) of HBc (upper left). In contrast, siRNA treatments against NXF1 and p15 (upper right), ALY and UIF (lower left), as well as BAT1 and DDX39 (lower right), induced nuclear accumulation of HBc protein. **B)** Three different subcellular distribution patterns of HBc are cartoon illustrated. N>C: nucleus predominant pattern; C>N: cytoplasm predominant pattern; N+C: present in both nucleus and cytoplasm, ratio N>C/C>N: tendency of nuclear accumulation. **C)** Quantitative results of the IFA assay in A) were summarized in the Table. Approximately 100–150 HBc-positive cells were scored in each transfection experiment. The data shown here represent an average from at least three independent transfections.

Next, we asked whether the physical associations between HBc, TREX components, and NXF1-p15 proteins (Fig. 2 and 3), have any functional significance in nuclear export of HBc protein. We perturbed the RNA export pathway by siRNAs against TREX components, such as ALY, UIF, BAT1/DDX39, and NXF1-p15 complex. As shown in the IFA (Fig. 4A), HBc protein was preferentially accumulated in the nucleus, when HBV-transfected HuH-7 cells were treated with siRNAs specific for NXF1 and p15 (*upper right panel*), ALY and UIF (*lower left panel*), or BAT1 and DDX39 (*lower right panel*). In contrast, HBc protein was localized predominantly in the cytoplasm, when HBV-transfected cells were treated with the non-target siRNA control (*upper left panel*, Fig. 4A). The results in Fig. 4A strongly suggest that HBc protein could utilize the TREX and NXF1-p15 RNA export pathway for nuclear exit. To analyze the IFA data in a more quantitative manner, we divided the HBc-positive cells into three different groups (N>C, C>N, and N+C), according to their patterns of subcellular distribution of HBc (Materials and Methods). We defined the “tendency of nuclear accumulation” as the ratio between numbers of cells with a nucleus-predominant pattern over those with a cytoplasm-predominant pattern (N>C/C>N) (Fig. 4B). The scored IFA data are summarized in the Table of Fig. 4C. Interestingly, despite the lack of detectable physical association between HBc and BAT1/DDX39 RNA helicase (Fig. 3C), reduction of BAT1/DDX39 by siRNA treatment did significantly arrest HBc protein in the nucleus (Fig. 4A and Fig. 4C).

The enrichment of nuclear HBc in Fig. 4 could be due to an off-target or indirect effect of the siRNA treatment. However, since the knockdown effect of siRNA treatments can be efficiently rescued by co-transfection with an expression vector of the host factors targeted by their respective siRNAs, the potential off-target effects of siRNA treatment are highly unlikely (data not shown). Another possibility is that nuclear enrichment of HBc was due to the reduced expression of a TREX-unrelated cellular factor that negatively regulated the nuclear localization of HBc. Similarly, a “co-depletion” or “cross-depletion” effect of the siRNA treatment has been reported in literature [40]–[42]. An siRNA-directed knockdown of one protein may also suppress the protein level of its complex partner protein, possibly because the complex loses its stoichiometry, integrity and stability. At present, a number of RNA export factors (NXF1, p15, ALY, UIF, BAT1, DDX39) are well known to be associated with TREX at different steps of the RNA export pathway [9]. It is noteworthy that ALL of these RNA export factors tested in Fig. 4 can invariably result in nuclear enrichment of HBc (6 out of 6, 100%). Therefore, it seems statistically unlikely that every knockdown of these RNA export factors happened to favor nuclear enrichment of HBc by some kind of indirect effects.

### Physical association between HBV pgRNA, TREX and the NXF1-p15 complex

**Figure 5:**
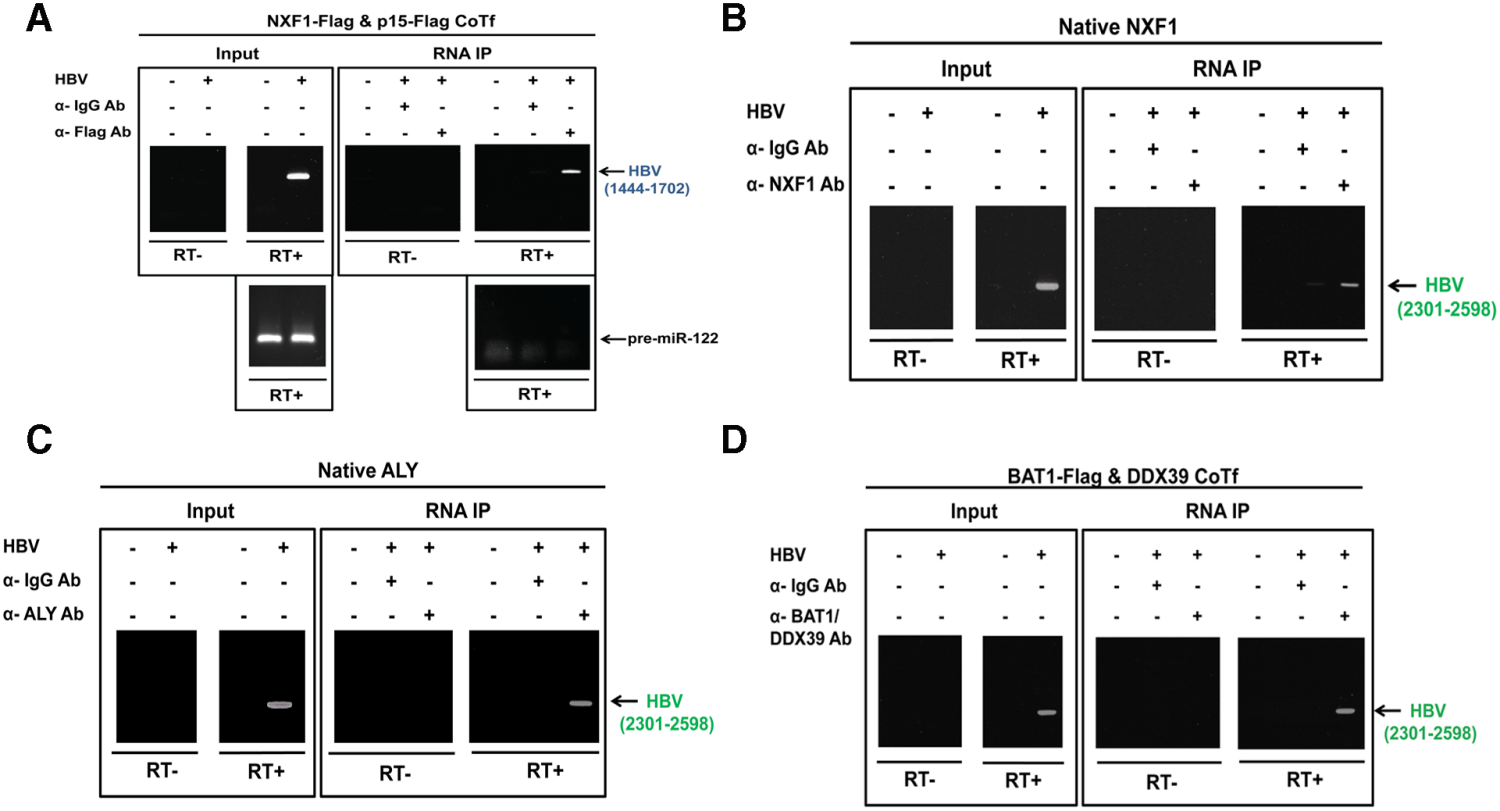
Physical association between HBV pgRNA, TREX and NXF1-p15 complex was revealed by the RNA-immunoprecipitation assay (RNA-IP). **A)** The NXF1-p15 complex can associate with HBV specific RNAs by the RNA-IP assay. HuH-7 cells were co-transfected with plasmid DNAs of an HBV genome, NXF1-Flag, and p15-Flag. HBV RNAs were extracted from immunoprecipitates using anti-Flag antibody, followed by RT-PCR analysis. PCR primers 1444 and 1702 can detect all species of HBV RNAs (Fig. 1). The most abundant pre-miR-122 in HuH-7 cells was used as a negative control RNA for its lack of physical association with NXF1-p15 complex. Anti-IgG antibody (α-IgG Ab) was included as a control for the specificity of immunoprecipitation. RT: reverse transcriptase. **B–D)** By the RNA-IP assay, HBV 3.5 kb non-spliced pgRNA was shown to be associated with several known protein components of the NXF1-p15 mediated RNA export machinery: **B)** NXF1, **C)** ALY, and **D)** BAT1/DDX39. PCR primers 2301 and 2598 can detect only the 3.5 kb non-spliced pgRNA (Fig. 1).

In a similar vein to our study of the HBc protein (Fig. 2–4), we asked whether HBV RNA can also utilize the NXF1-p15 machinery for nuclear export. To address this issue, we focused our study here on the 3.5 kb pgRNA, which serves as a template for HBV reverse transcription [4] as well as for translation of HBc and polymerase [3]. We examined whether HBV pgRNA can be physically associated with NXF1 protein by RNA immunoprecipitation (RNA-IP) assay (detailed in Materials and Methods). Briefly, HuH-7 cells were co-transfected with an HBV genome, NXF1-Flag, and p15-Flag plasmid DNAs. Transfected cell lysates were then incubated with anti-Flag antibody. Immunoprecipitated RNAs were then assayed by RT-PCR. As shown in Fig. 5A, the blue color-coded primer pair HBV 1444–1702 is included here as a positive control, since it can detect all the HBV specific RNA transcripts (Fig. 1). Nuclear export of microRNA precursors is known to be mediated by exportin-5 [43]. We therefore used a highly abundant pre-miR-122 as a negative control RNA for the lack of physical association with NXF1-p15 complex (Fig. 5A). Anti-Flag antibody can capture HBV RNAs, which can then be detected in a reverse transcriptase (RT)-dependent manner. On the other hand, control antibodies, such as anti-IgG antibody (Fig. 5A), as well as anti-tubulin antibody and anti-Rev antibody (data not shown), produced no significant HBV-specific RNA signals. Using this RNA-IP assay system established in Fig. 5A, we demonstrated that HBV pgRNA can be preferentially associated with NXF1 (Fig. 5B), ALY (Fig. 5C), and BAT1/DDX39 (Fig. 5D). One caveat here is that the interactions between RNA and RNA-binding proteins are known to be promiscuous in nature. Therefore, the RNA-IP assay may be limited in its specificity. As a side note, the green color-coded primer pair HBV 2301–2598 used in Fig. 5B–5D is relatively specific for the non-spliced pgRNA, since 1) it does not overlap with the HBx and HBsAg specific RNAs (Fig. 1); 2) the reverse primer 2598 is located within the intron of the major 2.2 kb spliced RNA (Fig. 1).

An HBV genome is known to encode a 3.5 kb precore specific RNA which is responsible for the production of precore and HBeAg proteins. This 3.5 kb precore transcript is longer than the 3.5 kb pgRNA by 25–30 nt at the 5′ terminus (Fig. 1). The rest of the precore RNA is essentially identical to that of the pgRNA. Precore RNA and its encoded precore and HBeAg proteins are not essential to HBV replication [44], [45]. The primer pair HBV 2301–2598 in theory cannot distinguish between the precore RNA and pgRNA. However, our HBV replicon system (plasmid pCHT-9/3091) contains a CMV promoter which does not produce the 3.5 kb precore specific RNA [30]. Therefore, HBV pgRNA, as detected by HBV primer pair 2301–2598, appeared to be physically associated with NXF1 and TREX complex (Fig. 5B–5D), both of which are important for the nuclear RNA export machinery [9].

### HBV pgRNA export facilitated by the NXF1-p15 machinery

**Figure 6:**
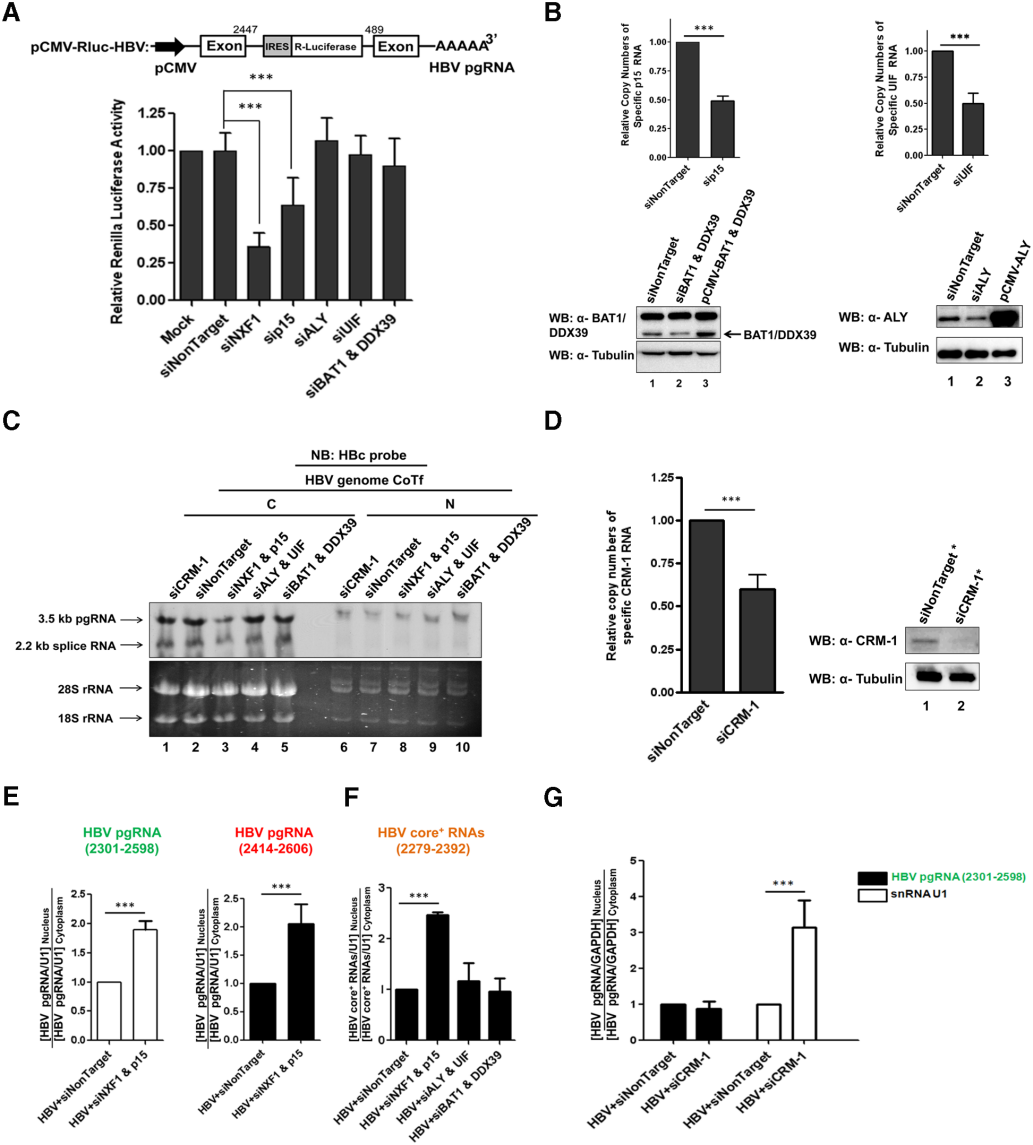
Both NXF1 and p15 can contribute to the efficient nuclear export of HBV pgRNA. **A)** *Upper panel:* A cartoon illustrates an HBV pgRNA reporter plasmid containing a Renilla luciferase gene inserted into a major intron of HBV pgRNA [25]. *Lower panel:* HuH-7 cells were co-transfected with an HBV pgRNA reporter plasmid and siRNAs. Reduction of the luciferase reporter activity was observed by treatment with siRNAs specific for NXF1-p15, but not with siRNAs specific for TREX components. To control for the indirect effect of siRNA knockdown (Material and Methods), Renilla luciferase activity was normalized with a co-transfected internal reference plasmid (firefly luciferase reporter). The relative Renilla luciferase activity of mock transfection was presented as 1. The graph here represents an average from at least three independent experiments. **B)** *Upper panel*: The knockdown efficacies of siRNAs specific for p15 and UIF were measured by RT-qPCR analysis, respectively. The copy number of RNA from siNonTarget treatment was presented as 1. *Lower panel:* Similarly, the efficacies of siRNAs specific for BAT1/DDX39 (lane 2, *left panel*) and ALY (lane 2, *right panel*) were measured by Western blot analysis, respectively. Expression vectors pCMV-BAT1, pCMV-DDX39 (lane 3, *left panel*), and pCMV-ALY (lane 3, *right panel*), were shown to produce their respective protein products by Western blot analysis. **C)** Only the siRNAs specific for NXF1 and p15 reduced the cytoplasmic core^+^ RNA levels by Northern blot (NB) analysis using an HBc specific probe (Fig. 1). Only weak signals of pgRNA were detected in the nuclear fraction. The faint signals of 45S, 32S and 20S ribosomal RNA precursors in the nuclear fraction can be seen after longer exposure [17]. **D)** The knockdown efficacy of siRNAs specific for CRM-1 was measured by RT-qPCR (left panel) and Western blot analyses (right panel). The copy number of RNA from siNonTarget treatment was shown as 1. *Transfected cells in the Western blot experiment were enriched by puromycin selection. The relative distribution of pgRNA (**E**) and HBV core^+^ RNAs (**F**) between the nuclear and cytoplasmic compartments was measured by RT-qPCR analysis. HBV RNAs were extracted from HuH-7 cells, which were co-transfected with an HBV genome and siRNAs specific for NXF1-p15, ALY and UIF, BAT1 and DDX39. Only the siRNA specific for NXF1 and p15 exhibited a higher N/C ratio of pgRNA, while no apparent effect was observed by siRNA specific for the TREX complex. The snRNA U1 was included as an internal control to normalize the N/C ratio. N/C: relative RNA levels between nucleus (N) and cytoplasm (C). The N/C ratio of HBV pgRNA or core^+^ RNAs from siNonTarget treatment was shown as 1. The graph represents an average from at least three independent experiments. **G)** Treatment with siRNA specific for CRM-1 resulted in no effect on the N/C ratio of HBV pgRNA. The snRNA U1, known to be exported by CRM-1 [49], was used as a positive control here. GAPDH was used as an internal control to normalize the N/C ratio. The N/C ratio of HBV pgRNA from siNonTarget treatment was shown as 1. The data here represent an average from at least three independent experiments.

Next, we performed a reporter assay for the functional study of HBV pgRNA export (Fig. 6A). The renilla luciferase reporter was inserted into the major intron of an HBV genome [25]. Therefore, only the non-spliced RNA in the cytoplasm can express the luciferase activity. As described in the legend of Fig. 6A, cell lysates of the reporter plasmid transfected HuH-7 cells, with or without siRNA treatment, were subject to the luciferase assay (Materials and Methods). The siRNAs specific for NXF1 or p15, but not TREX components (ALY, UIF, BAT1, and DDX39), resulted in significant reduction of the luciferase activity. The result in Fig. 6A suggests that the non-spliced reporter-containing RNA relies on the NXF1-p15 machinery for nuclear export. No apparent cytotoxicity from siRNA treatments was detected by the MTT assay (data not shown). The lack of effect from siRNA treatments specific for TREX components (ALY, UIF, BAT1 and DDX39), could be due to the functional redundancy between these TREX components and other cellular factors involved in the RNA processing and export machinery in the nucleus [46]. The efficacies of these siRNA treatments were monitored by RT-qPCR and Western blot analyses (Fig. 6B). The result of the reporter assay of Fig. 6A was confirmed by Northern blot analysis using an HBc specific probe, which does not overlap with HBs and HBx specific RNAs (Fig. 1). We found that the cytoplasmic levels of both pgRNA and the spliced 2.2 kb RNA were reproducibly reduced upon treatment with siRNA against NXF1 and p15 (compare lane 2 vs. lane 3, Fig. 6C), but not by siRNA treatments specific for TREX components (compare lane 4, 5 vs. lane 2, Fig. 6C). As mentioned in the Introduction, RNA export could also be mediated by another CRM-1 dependent pathway. As shown in Fig. 6C, no apparent change in pgRNA and 2.2 kb spliced RNA was observed by siRNA treatment specific for CRM-1 (compare lane 1 vs. lane 2, Fig. 6C). The efficacy of CRM-1 siRNA treatment was monitored by Western blot and RT-qPCR analyses (Fig. 6D). The reduction of cytoplasmic pgRNA by siRNA treatment specific for NXF1 and p15, suggests that the nuclear level of pgRNA should be elevated. However, we could detect only similar levels of weak HBV RNA signals in the nucleus by Northern blot analysis (Fig. 6C). It remains unclear to us what could be the explanation for this phenomenon. One possibility, which cannot be excluded, is the very rapid degradation of unsuccessfully exported mRNA by “nuclear exosome” of the nuclear RNA surveillance machinery [8], [47]. Other possibilities include the altered transcription of pgRNA and an indirect effect of the siRNA depletions. However, since the weak signals of pgRNA in the nucleus were similar between siRNA treatments specific for NXF1 or control siRNA, it is unlikely that cytoplasmic reduction of pgRNA was due to altered transcription.

We also took a direct measurement of HBV pgRNA in nuclear vs. cytoplasmic compartments using a quantitative RT-qPCR assay (Fig. 6E, *left panel*). Relative to the snRNA U1 control, the ratio of HBV specific pgRNA between the nucleus and cytoplasm was significantly and reproducibly increased upon treatment with siRNAs specific for NXF1 and p15. The result from green primer pair HBV 2301–2598 (Fig. 1; Fig. 6E, left panel) was confirmed by another red primer pair 2414–2606 (Fig. 1; Fig. 6E, right panel). We used snRNA U1 as a control here, since it was commonly used in literature as an internal reference for this type of assay [46], [48]. Consistent with the results in Fig. 6A and 6C, we observed no significant change in the relative distribution of core^+^ RNAs (Fig. 1) in nucleus vs. cytoplasm by siRNA treatments specific for ALY and UIF, or BAT1/DDX39 (Fig. 6F). As shown in Fig. 6G, siRNA treatment specific for CRM-1 had no apparent effect on pgRNA distribution between nucleus and cytoplasm, while snRNA U1 (known to be exported via a CRM-1 dependent pathway) was significantly increased in its nuclear accumulation (or cytoplasmic reduction) [49].

### HBc is not essential to HBV pgRNA nuclear export

**Figure 7:**
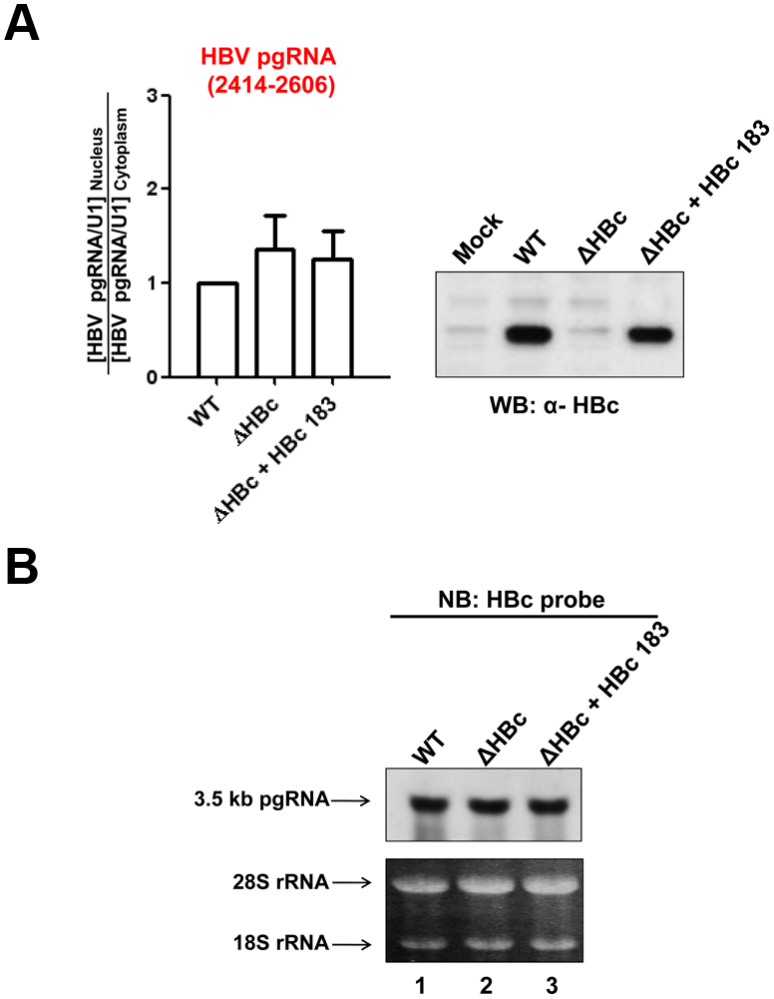
HBV core protein (HBc) exhibited no significant effect on nuclear export of the 3.5 kb pgRNA. **A)** HuH-7 cells were transfected with various plasmids of HBV genomes (based on plasmid pCHT-9/3091), containing wild type (WT) HBc, mutant ΔHBc (a core-deficient HBV genome), and a combination of ΔHBc and an expression vector of full-length HBc 183, respectively. HBV RNAs were extracted from nuclear and cytoplasmic compartments according to the Vendor’s protocol (Materials and Methods). Plasmid ΔHBc resulted in no apparent effect on the relative distribution of HBV pgRNA levels between nucleus and cytoplasm (N/C) by RT-qPCR analysis. The N/C ratio of HBV pgRNA from a WT HBV genome (plasmid pCHT-9/3091) was shown as 1. HBc protein expression was monitored by Western blot analysis (*right panel*). The graph represents an average from at least three independent experiments. **B)** Northern blot analysis revealed no significant reduction of the 3.5 kb pgRNA, in the cytoplasm of HuH-7 cells transfected with either a ΔHBc mutant or a combination of plasmids ΔHBc and HBc 183. Ribosomal RNA was included as an internal control for sample loading.

Since HBc can associate with RNA [50], as well as NXF1, p15, and ALY proteins (Fig. 2 and Fig. 3), we asked whether HBc could serve as an RNA-binding adaptor for HBV pgRNA nuclear export. We compared the relative distribution of HBV pgRNA between nucleus and cytoplasm by RT-qPCR analysis in the presence or absence of HBV core protein (Fig. 7A). The subcellular distribution of pgRNA appeared to be independent of wild type HBc, as detected by pgRNA specific primer pair 2414–2606 (Fig. 1). In this assay for the N/C ratio by RT-qPCR (Fig. 7A), HBV pgRNA in nucleus vs. cytoplasm was always normalized first with snRNA U1 as an internal control (or GAPDH, data not shown). Finally, by Northern blot analysis, we also detected no significant difference in the cytoplasmic HBV pgRNA levels, with or without HBc (Fig. 7B). In summary, HBV pgRNA nuclear export does not appear to require HBc protein.

### Potential influence of the NXF1-p15 complex on HBV DNA replication

**Figure 8:**
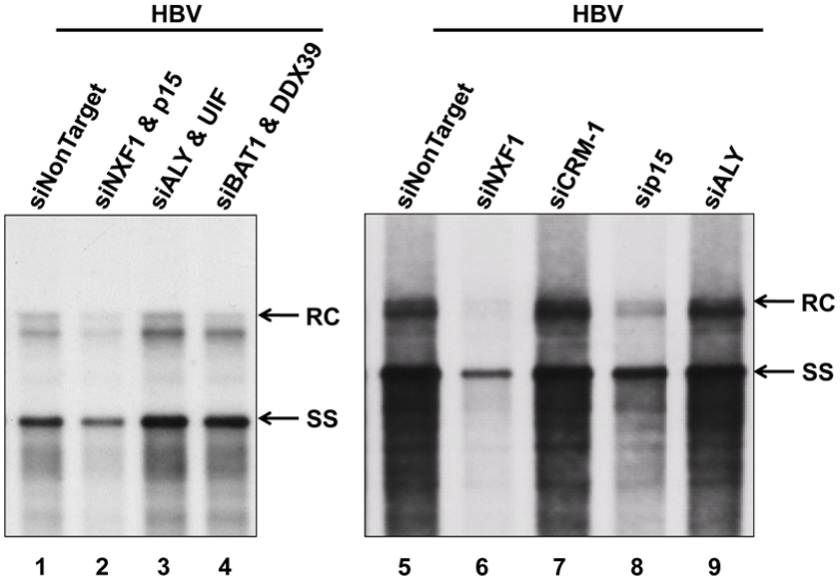
Interference with the NXF1-p15 complex reduced HBV DNA synthesis. HuH-7 cells were transiently co-transfected with an HBV genome (plasmid pCHT-9/3091) and siRNAs, followed by Southern blot analysis using HBV probe (nt 1521–3164) (Fig. 1). Upon treatment with siRNAs specific for p15 or NXF1-p15 complex, HBV replication was significantly inhibited. In contrast, no apparent effect on HBV replication was noted by treatment with various siRNAs specific for TREX components and CRM-1. RC: relaxed circle DNA, and SS: single-strand DNA.

Next, we asked whether perturbation of the NXF1-p15 pathway could influence HBV DNA replication. As shown in the Southern blot analysis of Fig. 8, treatments with siRNAs specific for NXF1 or p15, but not with siRNAs specific for TREX components and CRM-1, resulted in reproducible reduction of HBV DNA replication (lane 2, *left panel*; lane 6 and 8, *right panel,* Fig. 8). Most likely, the reduced HBV DNA synthesis was at least in part due to the reduced nuclear export of pgRNA (Fig. 6), when the efficiency of pgRNA nuclear export was compromised by the siRNA treatment specific for NXF1-p15.

## Discussion

**Figure 9:**
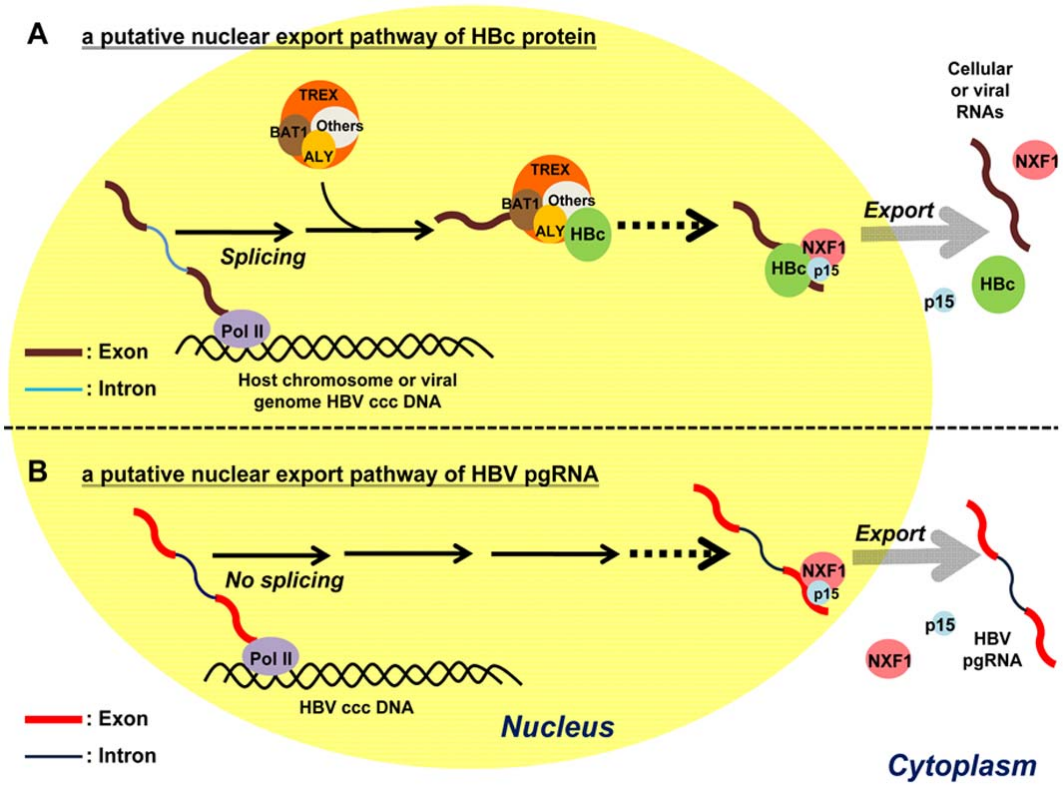
A graphic summary of the nuclear export of HBV pgRNA and HBc. **A)** A cartoon illustrates a putative nuclear export pathway of HBc protein. HBV DNA genome can exist episomally as a covalently closed circular form (ccc DNA) in the nucleus, which can serve as a template for pol-II mediated RNA transcription [3]. Pre-mRNA processing and nuclear export are known to be tightly coupled by the TREX complex, which consists of ALY, BAT1, and others (other known or unknown cellular factors) [65]. HBc protein can associate with ALY, but not BAT1. Dotted line indicates that the exact sequence or molecular mechanism remains unclear. HBc protein can also associate with NXF1 and p15. It is speculated here that the NXF1-p15 export machinery can recognize a stable RNP complex between HBc protein and RNA of either viral or cellular origins. **B)** Nuclear export of the 3.5 kb non-spliced pgRNA is dependent on the NXF1-p15 machinery. The exact molecular mechanism of pgRNA export remains to be further investigated in the future.

As summarized in Fig. 9, we illustrated putative nuclear export pathways of HBc protein (Fig. 9A) and pgRNA (Fig. 9B), respectively. We hypothesized here that HBc protein can get exported by usurping the cellular export machinery of spliced RNAs, including TREX and NXF1-p15 complex (Fig. 9A). Along the way to its nuclear export, HBc probably can form a messenger ribonucleoprotein (mRNP) complex with several RNA-binding adaptor proteins. These mRNP cargos can then target to nuclear pore complex (NPC) of the nuclear envelope before translocation into the cytoplasm. In contrast to the export of HBc protein, we have no evidence that the export of HBV pgRNA requires TREX, albeit it is dependent on NXF1-p15 (Fig. 9B).

### Intranuclear HBc protein and capsids

In the IFA experiments (Fig. 4), the anti-HBc antibody (*Dako, Denmark*) is of rabbit polyclonal origin. It can recognize HBc in monomeric, dimeric, and particulate forms. Similar IFA results were obtained by using an anti-HBc monoclonal antibody Hyb 3120 (*Institute of Immunology, Japan*) (data not shown). This antibody was shown to bind specifically to a conformational epitope of HBc capsid particles [51]–[53]. Therefore, it is very likely that the nuclear HBc protein could exist as partially or completely assembled capsid particles, instead of or in addition to HBc dimers [36].

### RNA can enhance HBc protein association with components of the RNA export machinery

In Fig. 2 and 3, physical associations between HBc or HBc ARD and NXF1, p15 or ALY, are RNase sensitive. However, even upon overnight treatment at higher concentrations of RNase, there were always residual protein-protein associations that appeared to be RNase resistant. A similar partially RNase sensitive and partially RNase resistant phenomenon of protein-protein association has been observed previously in other viral systems [16], [54], [55]. One interpretation for this “partially sensitive and partially resistant” phenomenon is that the RNase digestion was indeed already complete, yet some RNA-independent protein-protein association contributed to this phenomenon. In other words, RNA may enhance, but is not absolutely essential to, detectable protein-protein association. In the streptavidin pull down assay (Fig. 2E), neither the addition of RNase nor the total cellular RNA had any effect on the binding between HBc and NXF1-p15 (data not shown). How can one reconcile the results of RNase sensitivity in the co-IP experiment (Fig. 2A–2D) with the lack of effect of RNA in the streptavidin pull down assay (Fig. 2E and data not shown)? Recently, Viphakone et al. reported that TREX complex can provide a license for mRNA export by driving NXF1 into a conformation capable of binding RNA [56]. Therefore, it is possible that the RNA effect on protein-protein association cannot be detectable in the streptavidin pull down assay (Fig. 2E), unless all the essential components, including the TREX complex, is present.

### Nuclear export of HBc protein vs. pgRNA

Although treatments with siRNA specific for TREX components (e.g., ALY or BAT1/DDX39) could efficiently arrest HBc in the nucleus (Fig. 4), the same treatment did not inhibit pgRNA export (Fig. 6), or viral DNA replication (Fig. 8). Since HBc protein is not essential to pgRNA export (Fig. 7), it is not surprising that inhibition of HBc export by siRNA treatment did not have any apparent effect on pgRNA export and viral DNA synthesis. Functional redundancy between TREX component factors has been reported [46], [48], [57]. This phenomenon could explain the lack of any appreciable effect on pgRNA export by siRNA treatments specific for certain TREX components (Fig. 6). However, it remains a paradox that a similar treatment with siRNA specific for TREX components did arrest HBc in the nucleus (Fig. 4). There might be a subtle, yet important, difference in their respective requirements for nuclear export between HBc protein and pgRNA. On the other hand, differences between the interaction assays and the effects of depletion of factors by siRNA treatments, could in theory be attributed to a large number of reasons, such as instability of the complexes being measured during isolation, redundancy among the host factors, or indirect effects of factor depletion on cellular protein expression that participate in the event being measured.

### CRM-1 and PRE in HBV RNA splicing and export?

It is known that mRNA nuclear export can be mediated by CRM-1 or NXF1-p15 dependent pathway [9]. Previously, it has been studied that nuclear export of HBsAg specific RNAs can be facilitated by a so-called post-transcriptional regulatory element (PRE) (Fig. 1) [19]. It remains to be clarified whether PRE-mediated HBsAg RNA export is CRM-1 dependent [19], [58], [59] or independent (resistant to leptomycin B) [60]. In our current study, we have not investigated the relationship between PRE and the NXF1-p15 export pathway. However, reduction of CRM-1 (Fig. 6D) had no apparent effect on the subcellular distribution of pgRNA (Fig. 6C, 6G). Previous studies on PRE focused on the HBsAg RNAs [19], [20], [61], while our studies here focused on the full-length 3.5 kb pgRNA. It should also be noted here that PRE was reported to affect HBV RNA splicing, but no direct effect on pgRNA nuclear export [24]. PRE contains an intronic splicing silencer (ISS) which appears to repress alternative splicing in a sequence-independent, structure- and position-dependent manner [62]. Recently, a sub-element in PRE was reported to enhance nuclear export of intronless globin reporter mRNA by recruiting the TREX complex [63].

### Building up the pool size of ccc DNA and HBc protein shuttling

A surprising finding here is that the nuclear export of HBc protein appears to be facilitated by usurping the pathway of cellular mRNA export. The function of NES in HBc ARD domain could be mediated through the NXF1-p15 mRNA export mechanism. One potential biological significance in the intracellular trafficking of HBc is to recycle the HBc protein from nucleus to cytoplasm, and thus initiate another round of pgRNA encapsidation and reverse transcription in the cytoplasm. Repetitive rounds of HBc shuttling between nucleus and cytoplasm could help convert the relaxed circle (RC) DNA in mature capsids into covalently closed circular (ccc) DNA in the nucleus in DHBV infected hepatocytes [64]. Perturbation of HBV RNA export pathway could have a therapeutic potential in the future, if the accumulation of HBc in the nucleus results in no cytotoxicity or apoptosis to the host hepatocytes. Indeed, in clinical literature, nuclear accumulation of HBc protein tends to be associated with children and young adults with more benign and non-aggressive disease [27], [28]. Since the current treatment for hepatitis B patients often cannot effectively eradicate HBV cccDNA from hepatocytes, we entertain the idea of a potential therapeutic strategy by keeping HBc protein arrested in the nucleus, without achieving a molecularly sterile status of HBV DNA in hepatitis B patients.
